# Moderate- to vigorous-intensity physical activities for hemophilia A patients during low-dose pharmacokinetic-guided extended half-life factor VIII prophylaxis

**DOI:** 10.1186/s13023-024-03092-2

**Published:** 2024-03-26

**Authors:** Chonlatis Srichumpuang, Arunothai Rakmanotham, Chatphatai Moonla, Darintr Sosothikul

**Affiliations:** 1 Division of Pediatric Hematology and Oncology, Department of Pediatrics, Faculty of Medicine, Chulalongkorn University and King Chulalongkorn Memorial Hospital, Bangkok, Thailand; 2 Integrative and Innovative Hematology/Oncology Research Unit, Faculty of Medicine, Chulalongkorn University and King Chulalongkorn Memorial Hospital, Bangkok, Thailand; 3 Division of General Internal Medicine, Department of Medicine, Faculty of Medicine, Chulalongkorn University and King Chulalongkorn Memorial Hospital, Bangkok, Thailand; 4 Center of Excellence in Translational Hematology, Faculty of Medicine, Chulalongkorn University and King Chulalongkorn Memorial Hospital, Bangkok, Thailand

**Keywords:** Exercise, Factor VIII, Hemophilia A, Pharmacokinetics, Ruriroctocog alfa pegol

## Abstract

**Background:**

Low-dose pharmacokinetic (PK)-guided extended half-life (EHL) factor VIII (FVIII) prophylaxis can reduce the bleeding risk in hemophilia A (HA) patients. An increase in physical activities for promoting musculoskeletal health may enhance the benefits of prophylactic therapy.

**Objectives:**

To determine the clinical impact of moderate- to vigorous-intensity physical activities in HA patients during low-dose PK-guided EHL FVIII prophylaxis.

**Patients/Methods:**

This prospective study enrolled patients with moderate/severe HA (baseline FVIII levels ≤ 5 IU/dL) who had received low-dose PK-guided EHL FVIII prophylaxis for ≥ 6 months. An individualized exercise protocol was introduced to each participant, targeting a 65% increase in the maximum predicted heart rate for ≥ 150 min/week, while continuing low-dose PK-guided EHL FVIII prophylaxis for 6 months. Before and after implementing the intervention, annualized bleeding rates (ABR), annualized joint bleeding rates (AJBR), Hemophilia Joint Health Scores (HJHS), skeletal muscle mass, hemophilia-specific quality-of-life (QoL) scores and annualized FVIII consumption were compared.

**Results:**

Of 13 participants (mean age ± standard deviation [SD]: 20.1 ± 6.8 years), ABR, AJBR, and HJHS were significantly reduced (mean differences [MD] ± SD: −5.7 ± 2.6 bleeds/year, −4.2 ± 2.6 joint bleeds/year, and −4.3 ± 3.2 marks, respectively; *P* < 0.05) after applying the 6-month exercise protocol. Skeletal muscle mass and QoL scores had also improved (*P* = 0.001), while FVIII usage had decreased (MD ± SD: −129.1 ± 208.7 IU/kg/year; *P* < 0.05).

**Conclusions:**

The combination of moderate- to vigorous-intensity physical activities with low-dose PK-guided EHL FVIII prophylaxis improves bleeding prevention, musculoskeletal status and QoL in patients with moderate/severe HA. By minimizing FVIII consumption, this strategy helps optimize hemophilia care in countries with budget constraints. ClinicalTrials.gov NCT05728528.

**Supplementary Information:**

The online version contains supplementary material available at 10.1186/s13023-024-03092-2.

## Introduction

Hemophilia A (HA) is an X-linked recessive congenital bleeding disorder with a deficiency of coagulation factor VIII (FVIII). Patients with severe HA have endogenous FVIII coagulant activity (FVIII:C) levels < 1 IU/dL, making them vulnerable to spontaneous bleeding into joints and skeletal muscle, as well as the potential consequences of arthropathy and physical disability following repeated intraarticular bleeding [1]. Whereas in patients with moderate HA (FVIII:C levels 1–5 IU/dL), the bleeding phenotypes are variable but can be as severe as those with severe HA [2]. The current standard of care for patients with severe HA or moderate HA with a severe bleeding phenotype (annualized bleeding rate [ABR] ≥ 5 bleeds/year or annualized joint bleeding rate [AJBR] ≥ 3 joint bleeds/year) is a regular prophylaxis using FVIII concentrates [2, 3]. Prophylactic regimen with standard half-life (SHL) FVIII concentrates requires 2–4 intravenous infusions each week. Extended half-life (EHL) FVIII concentrates have been later developed using pegylation, albumin-fusion or immunoglobulin crystallizable fragment-fusion technologies to achieve 1.3–1.6 times longer half-life than SHL FVIII concentrates [4]. As a consequence, prophylaxis using EHL FVIII concentrates may allow reduction of FVIII infusions from thrice to twice weekly [5]. The efficacy and safety of EHL FVIII prophylaxis in reducing ABR, as compared to SHL FVIII prophylaxis, have been proven by several studies [6–9].

Different levels of physical activity or bleeding phenotypes among HA patients can affect the individual pharmacokinetic (PK) properties of FVIII concentrates. The variability in PK between individuals suggests that relying on a single average dose of FVIII concentrates may not optimize the prophylactic therapy for HA patients. Adjusting the infusion frequency and targeting a specific FVIII:C level based on the individualized PK characteristics would help personalize the prophylactic regimen [10]. A population-based PK (PopPK) software, myPKFiT® (Baxalta U.S. Inc., Lexington, MA), has been developed to support the optimal use of SHL recombinant FVIII concentrates (Advate®, Baxter Healthcare Corporation, Westlake Village, CA) and EHL recombinant FVIII concentrates (Adynovate®, Baxalta U.S. Inc., Lexington, MA) [11]. Recently, a study demonstrated the benefits and practicability of using low-dose EHL FVIII prophylaxis, guided by personalized PopPK data from myPKFiT®, in HA population [12].

In the past, HA patients were discouraged from participating in sports due to the perceived risk of sports-associated trauma and subsequent hemorrhages and morbidity [13]. However, recent studies have documented the physical, medical and psychosocial benefits of appropriate sports activities for HA patients. Supported by evidence that moderate- to vigorous-intensity physical activities or exercise transiently but significantly increase circulating endogenous FVIII:C and von Willebrand factor (VWF) levels, appropriate exercise may help diminish the bleeding risk in HA patients [14–15]. Besides, dynamic exercises are associated with improved muscle strength and joint health, as well as increased social inclusion and adaptation [16–17]. Although the World Federation of Hemophilia (WFH) recommends that patients with severe HA under FVIII prophylaxis should regularly perform appropriate physical activities or non-contact exercise, [3] data on this practice during low-dose FVIII prophylaxis is lacking. Therefore, this study aimed to investigate the impact of moderate- to vigorous-intensity physical activities in moderate/severe HA patients who receive low-dose PK-guided EHL FVIII prophylaxis, by comparing clinical outcomes between before and after implementing the appropriate exercise intervention.

## Methods

### Study population

This single-center non-randomized interventional study was conducted at King Chulalongkorn Memorial Hospital, Bangkok, Thailand, from January 2022 to December 2022. Participants were patients with severe HA (FVIII:C < 1 IU/dL) or moderate HA (FVIII:C 1–5 IU/dL) with a severe bleeding phenotype (ABR ≥ 5 bleeds/year or AJBR ≥ 3 joint bleeds/year), [2] aged at enrollment between 7 and 25 years, who had received low-dose PK-guided EHL FVIII prophylaxis for at least 6 months during the prior study [12]. Patients with comorbidities or conditions potentially affecting hemostasis or with the capability to not complete the study, such as detectable FVIII inhibitors at screening, planned major surgery, symptomatic human immunodeficiency virus infection, juvenile rheumatoid arthritis, metabolic bone diseases, or other conditions mimicking or causing joint diseases, were excluded. The study protocol was ethically approved by the Institutional Review Board, Faculty of Medicine, Chulalongkorn University (IRB no. 270/65; ClinicalTrials.gov. NCT05728528), and the written informed consent was obtained from each participant and their parents in accordance with the Declaration of Helsinki.

### Prophylactic therapy and exercise intervention

Each participant underwent an individual dose calculation of EHL Adynovate® by using PopPK software my-PKFiT®, as previously described, [12] and then received low-dose (10–20 IU/kg for 2–3 times/week) PK-guided EHL Adynovate® for at least 6 months before enrollment. Upon arrival at the hemophilia treatment center at the study site, eligible participants were approached and then evaluated for a complete medical history and physical examination, including body compositions measured by bioelectrical impedance analysis (BIA; InBody, InBody Co. Ltd., Seoul, South Korea), resting vital signs, and joint status. The baseline intensity of daily physical activities, including exercise, was determined by using the Global Physical Activity Questionnaire [18].

Sports medicine specialists analyzed the characteristics of each participant and designed individualized exercise protocols of moderate-to-vigorous intensity, targeting a 65% increase in the age-specific maximum predicted heart rate for at least 150 min/week or 60 min/day for 3 days/week, tailored based upon participant age, joint defects, and patient preference. Briefly, considering age-dependent capacity, exercise techniques requiring more muscle strength and coordination (e.g., wall push-ups, deadlifts) were selected for adolescents or adults, whereas less complicated techniques (e.g., 3-way coordination, slide movement) were appropriate for younger patients. For patients with the greater number of target joint(s), activities with higher movement or joint impact (e.g., running) were avoided, but techniques promoting endurance (e.g., treadmill walking) or flexibility and balance (e.g., tree pose, lateral leg raises) were more suitable in the designed programs. After introducing the full exercise protocol, each participant and their parents were asked to complete 6 exercise workshops (1 initial session followed by 5 additional monthly follow-up sessions) supervised by sports scientists. Each workshop included hemophilia-specific physical training and adjustments of exercise postures/techniques considering the current joint status and/or limitations after recent bleed(s). Participation in the assigned exercise program was monitored throughout the day using recorded heart rates and pace tracking via a smartwatch (Xiaomi, Xiaomi Inc., Beijing, China) equipped with an electrical sensor. Participants were obligated to report their daily physical activities to the investigator team via a mobile messenger application (LINE, Line Corporation, Tokyo, Japan).

In case of acute breakthrough bleeding, the investigator team would be promptly notified via the mobile application or by a direct telephone call. The exercise program was transiently interrupted while an intravenous dose of 10–20 IU/kg EHL Adynovate® was given immediately. Patients were closely observed; repeat doses of Adynovate® would be given every 12 h until the bleeding stopped.

### Study assessment and outcome measures

Data collected from the prior study [12] using low-dose EHL FVIII prophylaxis alone included ABR, AJBR, annualized amount of infused FVIII concentrates (for either regular prophylaxis or episodic treatment of breakthrough bleeds), and adherence to FVIII prophylaxis (percentage of actual to scheduled FVIII infusions) during the last 6 months. At enrollment of this study, joint status and number of target joints, hemophilia-specific health-related quality-of-life (QoL) scores, and body compositions were assessed. A target joint was defined as a joint with ≥ 3 spontaneous bleeds during a prior consecutive 6-month period [3]. Joint health status was determined by the Haemophilia Joint Health Score (HJHS) version 2.1 [19]. The total Haemo-QoL or Haemo-QoL-A questionnaires, depending on participant age, were used for QoL assessment [20–21]. A higher QoL score indicated a greater QoL impairment. All of these collected parameters were used as the pre-intervention data in this before-after study.

**Fig. 1:**
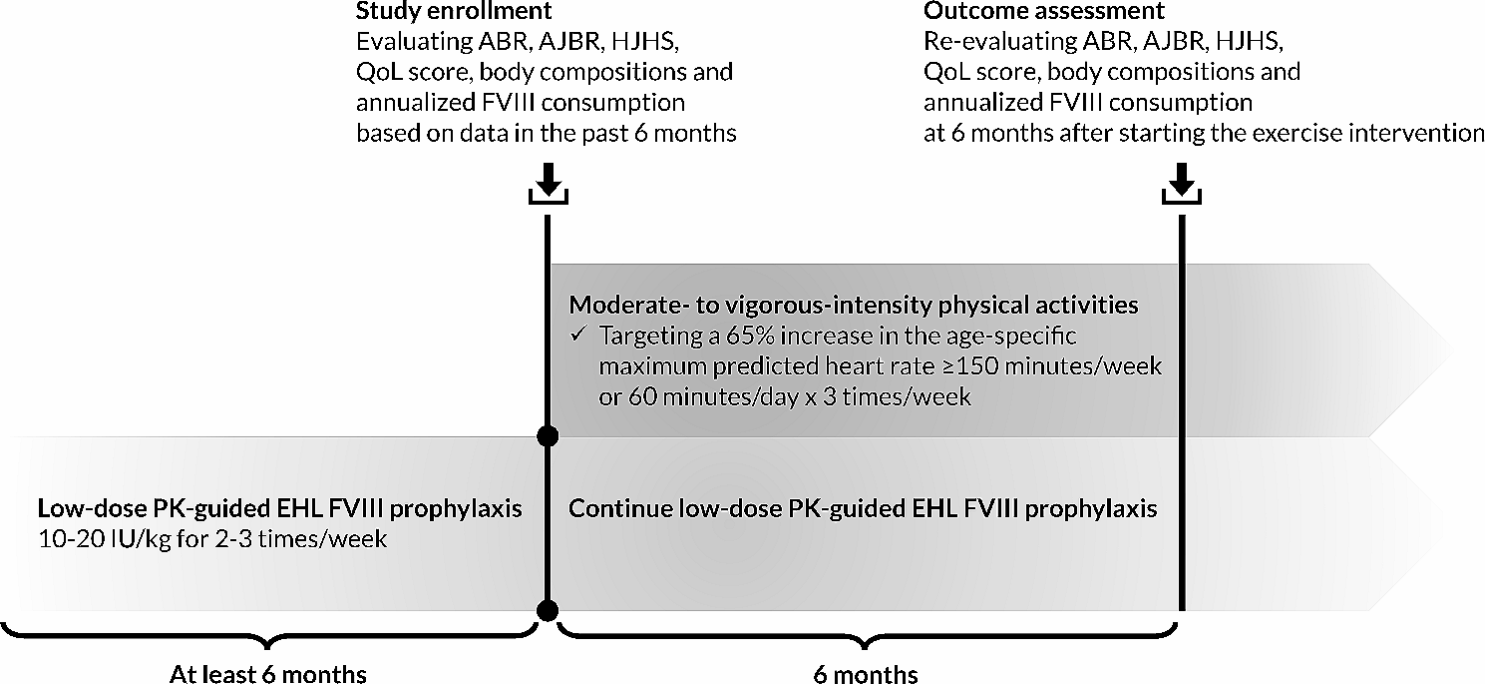
Study design. Abbreviations: ABR, annualized bleeding rate; AJBR, annualized joint bleeding rate; EHL, extended half-life; FVIII, factor VIII; HJHS, Hemophilia Joint Health Score; PK, pharmacokinetic; QoL, quality of life

The ABR, AJBR, HJHS and annualized FVIII consumption were prospectively evaluated after the 6-month period of moderate- to vigorous-intensity physical activities in combination with low-dose PK-guided EHL FVIII prophylaxis (Fig. 1). The pre- and post-intervention HJHS were independently assessed by two investigators (C.S. and D.S). Conflicts were resolved by mutual consensus among the investigators. Self-observed bleeding events, including hemarthrosis, were confirmed by the investigator team during the monthly follow-ups.

The primary outcome of the study was the bleeding rates, ABR and AJBR. The other hemophilia-related clinical outcomes, i.e., HJHS and skeletal muscle mass, adherence to the prophylactic regimen, QoL scores, and annualized FVIII consumption were secondary outcomes. All outcomes before and after the 6 months exercise intervention while continuing the same low-dose PK-guided EHL FVIII prophylaxis were compared.

### Statistical analysis

The Statistical Product and Service Solutions (SPSS) software version 22.0 (SPSS Inc., Chicago, IL) was used for all statistical analyses and GraphPad Prism version 9.5.1 (GraphPad Software Inc., Boston, MA) was used for figure customization. Categorical variables were presented as frequencies and percentages, while continuous variables were presented as median with interquartile range (IQR) or mean with standard deviation (SD). Outcome comparisons were performed using Wilcoxon signed-rank test and paired *t* test. The significant level was set at *P* < 0.05.

Subgroup analyses based on age at enrollment (≤ 15 vs. >15 years) and the presence of target joint(s) were prespecified. These assumptions were derived from the prior study [12] and the observations that (1) joint health in younger patients tended to be better and (2) target joint(s) increased the risk of spontaneous hemarthrosis in which higher trough FVIII:C levels were probably needed for FVIII prophylaxis.

## Results

### Patients and baseline characteristics

Of 15 eligible candidates, 13 patients were enrolled in the study. Two patients declined to participate due to residing far from the study site and the inconvenience of frequent follow-up during the COVID-19 pandemic. The baseline characteristics of 13 participants are outlined in Table 1. The mean participant age (SD) was 20.1 (6.8) years, and 11 patients (84.6%) had severe HA. All patients had previously received regular FVIII prophylaxis. In 4 patients aged ≤ 15 years (30.8%), 3 and 1 patients used primary and secondary prophylaxis, respectively. Whereas in 9 patients age > 15 years (69.2%), 8 patients used tertiary prophylaxis, but 1 patient used secondary prophylaxis. During 6 months before this study, 8 and 5 patients infused EHL FVIII concentrates twice and thrice weekly, respectively, as part of their low-dose PK-guided EHL FVIII prophylaxis. Individual PK parameters and PK-guided prophylactic regimens are described in Supplementary Table S1.

**Table 1:** Baseline characteristics of the study participants (*N* = 13)

Patient characteristics	Statistical values
No. of participants, *N*	13
Age in years, mean (SD)	20.1 (6.8)
Natural baseline FVIII:C levels, *n* (%)	
• < 1 IU/dL	8 (61.5)
• 1–3 IU/dL	5 (38.5)
ABR while using low-dose PK-guided EHL FVIII prophylaxis before study inclusion, bleeds/year	
• Mean (SD)	9.5 (7.1)
• Median (IQR)	10 (3–14)
AJBR while using low-dose PK-guided EHL FVIII prophylaxis before study inclusion, joint bleeds/year	
• Mean (SD)	6.7 (7.3)
• Median (IQR)	4 (0–12)
HJHS while using low-dose PK-guided EHL FVIII prophylaxis before study inclusion, marks	
• Mean (SD)	16.5 (12.3)
• Median (IQR)	14 (10–24)
No. of target joint(s), *n* (%)	
• None	3 (23.1)
• 1 joint	1 (7.7)
• 2 joints	2 (15.4)
• 3 joints	7 (53.8)
Annualized FVIII consumption while using low-dose PK-guided EHL FVIII prophylaxis before study inclusion, IU/kg/year	
• Mean (SD)	1581.5 (430.6)
• Median (IQR)	1724.8 (1235.4–1778.5)

Abbreviations: ABR, annualized bleeding rate; AJBR, annualized joint bleeding rate; EHL, extended half-life; FVIII, factor VIII; FVIII:C, factor VIII coagulant activity; HJHS, Hemophilia Joint Health Score; IQR, interquartile range; IU, international unit; PK, pharmacokinetic; SD, standard deviation.

Among 10 patients (76.9%) with target joint(s), 9 patients had ≥ 2 target joints; 8 patients used tertiary prophylaxis while 2 patients used secondary prophylaxis. While the other 3 patients (23.1%) without a target joint had received primary prophylaxis since early childhood. The ABR, AJBR, and HJHS during the 6-month pre-study period are shown in Table 1. Before initiating the exercise protocol, the median annualized consumption of EHL FVIII concentrates, including dosages for regular prophylaxis and on-demand treatment of breakthrough bleeds, was 1724.8 IU/kg/year (IQR 1235.4−1778.5). A moderate- to vigorous-intensity physical activity program designed by sports medicine specialists was assigned to each patient as outlined in Supplementary Table S2.

### Bleeding outcomes

**Fig. 2:**
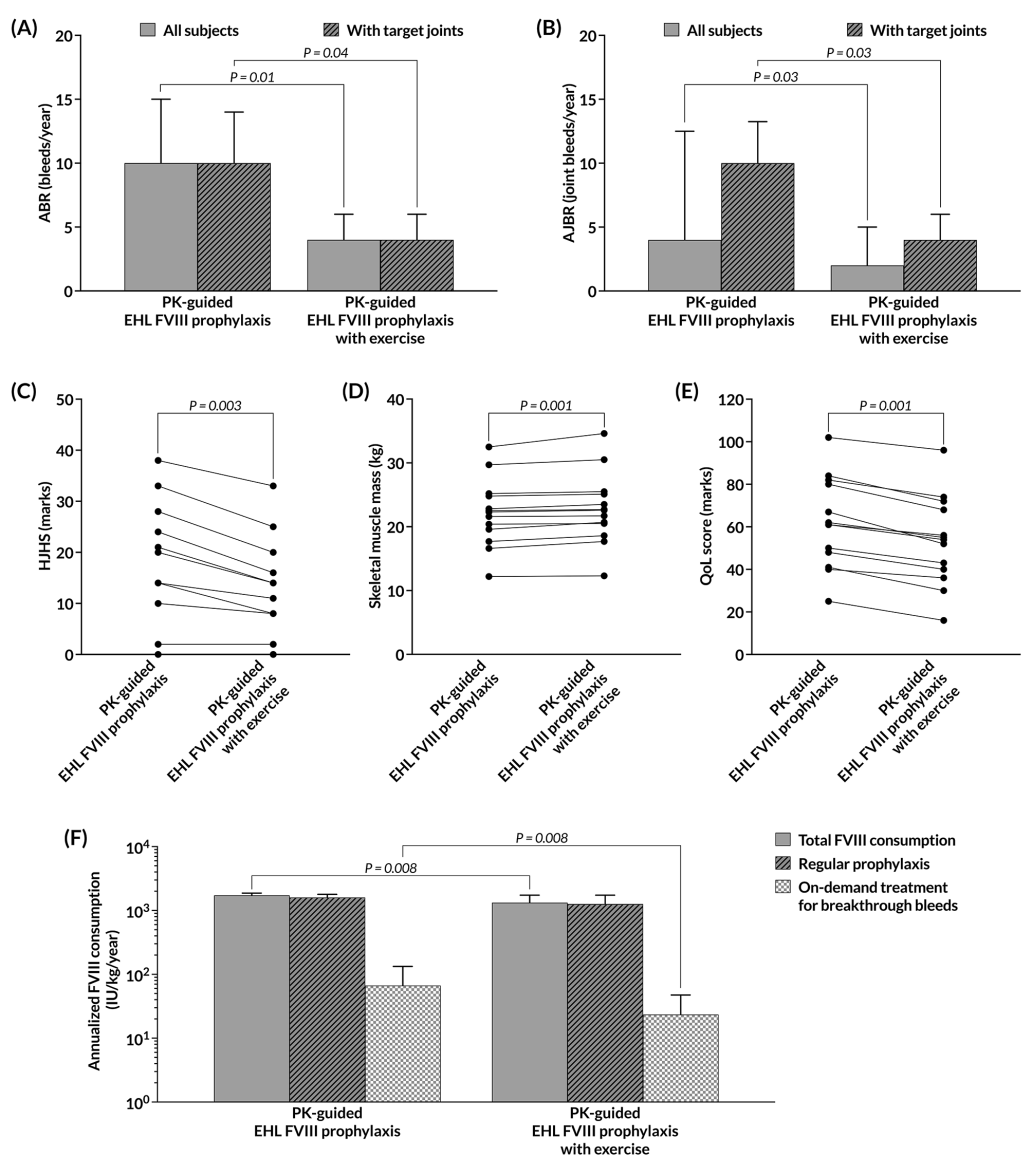
Changes in (**A**) ABR, (**B**) AJBR, (**C**) HJHS, (**D**) skeletal muscle mass, (**E**) QoL scores and (**F**) annualized FVIII consumption between before and after the 6-month exercise intervention. Abbreviations: ABR, annualized bleeding rate; AJBR, annualized joint bleeding rate; EHL, extended half-life; FVIII, factor VIII; HJHS, Hemophilia Joint Health Score; IU, international unit; PK, pharmacokinetic; QoL, quality of life

Six months after implementing the exercise protocol, in combination with low-dose PK-guided EHL FVIII prophylaxis, the ABR and AJBR were reduced significantly (Table 2; Fig. 2A and B). The median (IQR) ABR and AJBR of 10 (3–14) bleeds/year and 4 (0–12) joint bleeds/year were decreased to 4 (2–6; *P* = 0.01) bleeds/year and 2 (0–4; *P* = 0.03) joint bleeds/year, respectively. The mean differences (SD) in ABR and AJBR were −5.7 (2.6) bleeds/year (*P* = 0.005) and −4.2 (2.6) joint bleeds/year (*P* = 0.02). In the subgroups of age > 15 years or with target joint(s), decrease in the median AJBR also reached statistical significance (Table 2). Notably, 6 patients (46.2%) were able to achieve zero joint bleeds; 3 of them had target joint(s).

**Table 2:** Hemophilia-related clinical outcomes before and 6 months after implementing moderate- to vigorous-intensity physical activity protocol during low-dose PK-guided EHL FVIII prophylaxis

Outcome parameters	Statistical values, median (IQR)		***P***-value
	Before study inclusion	6 months after the start of the exercise intervention	
**ABR**			
All patients (*N* = 13)	10 (3–14)	4 (2–6)	0.01
Subgroup by age			
≤ 15 years (*n* = 4)	9 (1–16)	2 (0–5)	0.09
> 15 years (*n* = 9)	10 (4–14)	4 (4–6)	0.049
Subgroup by target joints			
Present (*n* = 10)	10 (3–14)	4 (2–6)	0.04
Absent (*n* = 3)	16 (0–16)	4 (0–6)	0.17
**AJBR**			
All patients (*N* = 13)	4 (0–12)	2 (0–4)	0.03
Subgroup by age			
≤ 15 years (*n* = 4)	0 (0–0)	0 (0–0)	NS
> 15 years (*n* = 9)	10 (4–13)	4 (2–6)	0.03
Subgroup by target joints			
Present (*n* = 10)	10 (2–13)	4 (0–6)	0.03
Absent (*n* = 3)	0 (0–0)	0 (0–0)	NS
**HJHS**			
All patients (*N* = 13)	14 (10–24)	11 (8–16)	0.003
Subgroup by age			
≤ 15 years (*n* = 4)	1 (0–6)	1 (0–5)	0.32
> 15 years (*n* = 9)	21 (14–28)	14 (11–20)	0.007
Subgroup by target joints			
Present (*n* = 10)	20.5 (14–28)	14 (8–20)	0.005
Absent (*n* = 3)	0 (0–2)	0 (0–2)	0.99
**QoL scores**			
All patients (*N* = 13)	61(48–80)	54 (40–68)	0.001
Subgroup by age			
≤ 15 years (*n* = 4)	55.5 (37.5–72.5)	49.5 (29.5–64)	0.01
> 15 years (*n* = 9)	62 (48–80)	54 (40–68)	0.008
Subgroup by target joints			
Present (*n* = 10)	61.5 (41–80)	53 (36–68)	< 0.001
Absent (*n* = 3)	61 (50–84)	56 (43–72)	0.06
**Skeletal muscle mass (kg)**			
All patients (*N* = 13)	22.3 (19.6–24.8)	22.6 (20.5–25.1)	0.001
Subgroup by age			
≤ 15 years (*n* = 4)	18.1 (14.4–26.1)	19.2 (15.0–27.7)	0.06
> 15 years (*n* = 9)	22.5 (21.6–24.8)	22.7 (21.7–25.1)	0.008
Subgroup by target joints			
Present (*n* = 10)	22.7 (21.6–25.2)	23.1 (21.7–25.5)	0.005
Absent (*n* = 3)	16.6 (12.2–19.6)	17.7 (12.3–20.7)	0.10

Abbreviations: ABR, annualized bleeding rate; AJBR, annualized joint bleeding rate; HJHS, Hemophilia Joint Health Score; IQR, interquartile range; NS, not significant; QoL, quality of life.

### Musculoskeletal status, adherence to prophylactic therapy and quality of life

The median (IQR) HJHS of 14 marks (10–24) was improved to be 11 marks (8–16) after the 6-month exercise intervention (*P* = 0.003; Table 2; Fig. 2C). The mean difference (SD) in HJHS was −4.3 (3.2) marks (*P* = 0.001). The significant changes were observed in the subgroups of age > 15 years or with target joint(s) (Table 2). A lower HJHS indicated heathier joints in terms of joint swelling, pain in motion, limited range of motion and loss of muscle power due to pain.

The body compositions were measured by BIA in all participants. The significant increase in skeletal muscle mass was observed after the 6-month exercise intervention, with the mean difference (SD) of 0.62 (0.58) kg (*P* = 0.001; Table 2; Fig. 2D), especially in the subgroups of age > 15 years or with target joint(s). The adherence to low-dose PK-guided EHL FVIII prophylaxis during the study period (median 91% [IQR 86–94%]) was not significantly different from that during the 6-month pre-study period (median 88% [IQR 85–95%]). Comparing between before and after implementing the exercise intervention, the QoL scores showed significant improvement (median [IQR] 61 [48–80] vs. 54 [40–68] marks; *P* = 0.001; Table 2; Fig. 2E).

### Factor consumption

Compared to the 6-month pre-study period, the total amount of infused FVIII concentrates, used for regular prophylaxis and on-demand treatment of breakthrough bleeds, was significantly decreased after the 6-month exercise intervention (*P* = 0.008; Table 3), with the mean difference (SD) of −129.1 (208.7) IU/kg/year (*P* = 0.046). Specifically, the factor consumption for on-demand treatment, not for regular prophylaxis, was reduced (Fig. 2F). The significant reduction was apparently demonstrated in the subgroups of age > 15 years or with target joint(s).

**Table 3:** The annualized factor consumption

**Patients and subgroups**	**Total factor consumption (IU/kg/year), median (IQR)**		P-value
	Before study inclusion	6 months after the start of the exercise intervention	
All patients (N = 13)	1724.8 (1235.4-1778.5)	1322.1 (1258.7-1727.6)	0.008
Subgroup by age			
≤ 15 years (n = 4)	1753.0 (1351.0-2078.0)	1736.3 (1336.5–2028.0)	0.09
> 15 years (n = 9)	1514.4 (1235.4-1764.7)	1307.5 (1258.7-1601.6)	0.02
Subgroup by target joints			
Present (n = 10)	1403.6 (1095.3-1764.7)	1284.6 (1067.8-1601.6)	0.01
Absent (n = 3)	1778.5 (1727.6-2377.8)	1745.0 (1727.6-2311.1)	0.17

Abbreviations: IQR: interquartile range; IU: international unit.

## Discussion

This prospective cohort study reveals the significantly positive effects of moderate- to vigorous-intensity physical activities on regular FVIII prophylaxis using low-dose PK-guided EHL FVIII concentrates among Thai patients with moderate/severe HA. The prophylaxis-exercise combination can reduce ABR and AJBR, as well as improve HJHS and QoL scores. Not only does it improve the joint status, but it also increases skeletal muscle mass, which potentially strengthens the overall physical abilities and performance of HA patients. Furthermore, the decreased annualized FVIII consumption, related to lower breakthrough bleeding events, may help decrease the cost of treatments in countries with budget constraints [22]. Incorporating an individualized non-contact exercise program of increased intensity during the appropriate FVIII prophylaxis supports optimizing hemophilia management, which currently aims for near-zero-bleed prevention along with maximization of QoL improvement, in the HA population.

In a prior study, [12] switching from low-dose weight-based SHL FVIII prophylaxis to low-dose PK-guided EHL FVIII prophylaxis resulted in significant decreases in ABR, AJBR and HJHS in patients with moderate/severe HA, especially in the subgroups of age > 15 years or with target joint(s). However, no participants in these subgroups achieved zero joint bleeds. Although target joints were usually found in adults with HA who had not received primary or secondary prophylaxis since childhood and had inadequate tertiary prophylaxis, and acute/subacute joint outcomes (i.e., joint swelling and joint pain on motion) could be improved after using low-dose PK-guided EHL FVIII prophylaxis for at least 6 months [12], chronic joint outcomes (i.e., range of motion, crepitus, muscle strength, and muscle atrophy) still persisted. On the contrary, those in the subgroups of age ≤ 15 years or without a target joint tended to initiate FVIII prophylaxis earlier, leading to more preferable outcomes [12].

Several randomized and non-randomized studies have reported that programmed exercise therapy, including aquatic sports and treadmill walking, could improve muscle strength, range of motion, overall joint functions and HJHS, without increasing the bleeding risk in HA patients [13]. Nevertheless, a FVIII:C level of at least 8–11 IU/dL may be required during exercise to avoid sports-induced bleeds [23]. In this study, despite the target joints presented in 76.9% of the study population, we observed that applying moderate- to vigorous-intensity physical activities, coupled with low-dose PK-guided EHL FVIII prophylaxis, not only benefits musculoskeletal status but also enhances bleeding prevention, resulting in zero joint bleeds in 46.2% of the patients. Regular exercises, thoughtfully designed to suit age and baseline joint status, are potentially safe for patients with moderate/severe HA when receiving adequate FVIII prophylaxis. Supported by previous evidence, [14, 15] engaging in exercise sessions throughout the week may contribute to pulsatory procoagulant effects on overall hemostasis, thus improving bleeding control, even in the subgroup with target joint(s).

The challenges in this combined practice were how to monitor the adherence to FVIII prophylaxis and the compliance of participating in the exercise program. An individual approach using new digital technologies, i.e., smartwatch monitoring and mobile messenger application, facilitated the retrieving of updated patient data in real-time and promptly resolving any obstacles that would occur during the program. This method also helped support home-based consultation and promoted mutual relationship between the hemophilia care team and HA patients and/or their families. Our experience suggests that these telemedicine technologies should be considered for assisting the prophylaxis-exercise strategy in the real-world situation. Better adherence to treatments would lead to a nearly normal life and a better QoL in HA patients. As a result of the reduction in ABR and AJBR following the 6-month exercise intervention, the total annualized FVIII consumption had correspondingly decreased due to a decline in FVIII replacement therapy required for breakthrough bleeds. Nonetheless, the amounts of FVIII concentrates primarily used for regular prophylaxis remained unchanged. These findings thus encourage HA patients under adequate low-dose FVIII prophylaxis to participate in more active physical activities. Not only does it help reduce FVIII consumption, but it also optimizes hemophilia care, particularly in resource-constrained circumstances, by minimizing the cost of treatments [22].

Our study has limitations. First, the sample size was small due to a single-center study design, the restricted inclusion criteria, and the requirement of full engagement in both prophylaxis and exercise interventions from the participants. Future large, multicenter studies are warranted to provide more affirmative evidence of the advantages on clinical outcomes. Second, most of the patients were older than 15 years, had target joint(s), and received tertiary FVIII prophylaxis. The benefits on ABR and AJBR might be largely influenced by the improvement of joint health status. Therefore, the generalizability of data to pediatric patients with HA using primary or secondary FVIII prophylaxis would be limited. Third, our study did not evaluate changes in any hemostatic parameters, i.e., VWF levels, thrombin generation and clot formation [14, 15]. Long-term effects on these hemostatic properties and mean trough FVIII:C levels in relation to intensities of physical activities that may support our findings remain to be determined. Fourth, only low-dose PK-guided EHL FVIII prophylaxis was used in this study. Hence, the application of the prophylaxis-exercise strategy using low-dose weight-based SHL FVIII prophylaxis should be done with caution.

## Conclusions

This is the first prospective clinical study conducted in Thailand to integrate moderate- to vigorous-intensity physical activities into patients with moderate/severe HA who received low-dose PK-guided EHL FVIII prophylaxis. Despite the relatively small sample size, this approach enhances the benefits of prophylactic therapy by improving bleeding prevention, musculoskeletal status, and hemophilia-specific health-related QoL in the Thai HA population. Participating in appropriate physical activities or exercise under adequate low-dose EHL FVIII prophylaxis may reduce the risk of bleeding and improve joint health, especially in patients with target joints. Individualized management that combines regular prophylactic therapy with exercise of increased intensity holds potential as part of the standard of care for HA and should be accompanied by study plans to further investigate its effectiveness in either clinical or surrogate outcomes in hemostasis.

### Electronic supplementary material

Below is the link to the electronic supplementary material.

Supplementary Material 1

## Data Availability

C.S. and D.S. have full access to all data and take responsibility for the integrity and accuracy of data. All data supporting the findings of this study are available within the paper and its Supplementary Information. Raw data are available from the corresponding author upon reasonable request.
