# Supplementary material for: Moderate- to vigorous-intensity physical activities for hemophilia A patients during low-dose pharmacokinetic-guided extended half-life factor VIII prophylaxis
Source: Orphanet J Rare Dis. 2024 Mar 26;19:135. doi: 10.1186/s13023-024-03092-2 (PMC10967220; doi:10.1186/s13023-024-03092-2)
Supplement: Supplementary file 1 — Supplementary Material 1 [file 13023_2024_3092_MOESM1_ESM.docx]

**Supplementary Appendix**

Supplement to: Srichumpuang C, Rakmanotham A, Moonla C, Sosothikul D. Moderate- to vigorous-intensity physical activities for hemophilia A patients during low-dose pharmacokinetic-guided extended half-life factor VIII prophylaxis. 2023.

This supplemental material has been provided by the authors to give readers additional information about their work.

**Table of Contents**

**Page**

**Supplementary Table S1** S2

Individualized pharmacokinetics (PK) properties and low-dose PK-guided extended half-life factor VIII prophylactic regimen used by the study participants.

**Supplementary Table S2** S3

Moderate- to vigorous-intensity physical activities assigned to the study participants.

**Supplementary Table S1** Individualized pharmacokinetics (PK) properties and low-dose PK-guided extended half-life factor VIII prophylactic regimen used by the study participants.

| **No.** | **Age (years)** | **Fat free mass (kg)** | **Bioavailability of EHL FVIII (PK-guided calculation)** | | | **Target joint(s)** | **Low-dose PK-guided**  **EHL FVIII concentrates**  **(IU/dose in a week)** |
| --- | --- | --- | --- | --- | --- | --- | --- |
|  |  |  | **Clearance (dL/hr/kg)** | **FVIII:C half-life (hr)** | **Time below 1 IU/dL (hr)** |  |  |
| 1 | 18 | 41.3 | 0.016 | 15.5 | 16 | Left ankle, both hips | 500, 500 |
| 2 | 20 | 48.7 | 0.017 | 14.7 | 0 | Right knee, right ankle | 500, 500, 500 |
| 3 | 11 | 30.1 | 0.016 | 18.9 | 0 | None | 500, 500 |
| 4 | 26 | 47.6 | 0.030 | 11.0 | 23 | Both elbows, left knee | 500, 500, 500 |
| 5 | 26 | 42.9 | 0.022 | 15.2 | NE | Left ankle | 500, 500 |
| 6 | 24 | 48.7 | 0.028 | 13.7 | NE | Both hips, left ankle | 500, 500 |
| 7 | 7 | 27 | 0.018 | 12.6 | NE | None | 500, 500 |
| 8 | 24 | 49.3 | 0.017 | 19.0 | 10 | Right elbow, left knee, right ankle | 500, 500 |
| 9 | 26 | 64.2 | 0.034 | 13.4 | 46 | Right knee, Left ankle | 500, 500, 500 |
| 10 | 26 | 42.7 | 0.035 | 11.4 | 22 | Both elbows, both knees, right ankle | 500, 500, 500 |
| 11 | 24 | 41.6 | 0.028 | 13.1 | 51 | Both knees, left ankle | 500, 500 |
| 12 | 13 | 29.8 | 0.027 | 12.0 | 45 | None | 500, 500 |
| 13 | 14 | 55 | 0.025 | 15.2 | 11 | Right ankle, both elbows | 500, 500 |
| Abbreviations: EHL, extended half-life; FVIII, factor VIII; FVIII:C, factor VIII coagulant activity; PK, pharmacokinetics; NE, not estimable. | | | | | | | |

**Supplementary Table S2** Moderate- to vigorous-intensity physical activities assigned to the study participants.

| **Day of each week** | **Group^†^** | **Exercise protocol** | | | | |
| --- | --- | --- | --- | --- | --- | --- |
|  |  | **Warm up** | **Work out** | **Cool down** | **Note** |  |
| 1 | 1 | Stretching | **Body weight:** wall squats, glute bridge, calf raises, full plank | Stretching | Duration 30 minutes/day |  |
|  | 2 |  | **Body weight:** wall squats, glute bridge**,** calf raises, full plank**,** wall push-ups, Hamstring curl  (15 seconds/set for each exercise posture, 6 sets for each exercise posture) |  | Duration 30-45 minutes/day |  |
|  | 3 |  | **Body weight:** wall squats, glute bridge**,** calf raises, full plank**,** wall push-ups, Hamstring curl  (15 seconds/set for each exercise posture, 6 sets for each exercise posture) |  | Duration 30-45 minutes/day or as tolerated |  |
| 2 | 1 |  | **Freestyle activity (biking, running)** |  |  |  |
|  | 2 |  | **Freestyle activity (biking, swimming)** |  |  |  |
|  | 3 |  | **Freestyle activity (biking, swimming)** |  |  |  |
| 3 | 1 |  | **Movement:** 3-way coordination, slide movement, in and out exercise, high knees |  | Duration 30 minutes/day |  |
|  | 2 |  | **Endurance:** treadmill walking, high knees, step jumping jacks, air punch (10 minutes/set for each exercise posture, 3 sets for each exercise posture) |  | Duration 30-45 minutes/day |  |
|  | 3 |  | **Endurance:** treadmill walking, high knees, step jumping jacks, air punch (10 minutes/set for each exercise posture, 3 sets for each exercise posture) |  | Duration 30-45 minutes/day or as tolerated |  |
| 4 | 1 |  | **Freestyle activity (biking, running)** |  |  |  |
|  | 2 |  | **Freestyle activity (biking, swimming)** |  |  |  |
|  | 3 |  | **Freestyle activity (biking, swimming)** |  |  |  |
| 5 | 1 |  | **Flexibility & Balance:** toe touch, tree pose, knee to chest, cobra pose |  | Duration 30 minutes/day |  |
|  | 2 |  | **Flexibility & Balance:** deadlifts, tree pose, lateral leg raises, Around the world (1 minute/set for each exercise posture, 5 sets for each exercise posture) |  | Duration 30-45 minutes/day |  |
|  | 3 |  | **Flexibility & Balance:** deadlifts, tree pose, lateral leg raises, Around the world (1 minute/set for each exercise posture, 5 sets for each exercise posture) |  | Duration 30-45 minutes/day or as tolerated |  |
| 6 | 1 |  | **Freestyle activity (biking, running)** |  |  |  |
|  | 2 |  | **Freestyle activity (biking, swimming)** |  |  |  |
|  | 3 |  | **Freestyle activity (biking, swimming)** |  |  |  |
| 7 | 1 |  | **Freestyle activity (biking, running)** |  |  |  |
|  | 2 |  | **Freestyle activity (biking, swimming)** |  |  |  |
|  | 3 |  | **Freestyle activity (biking, swimming)** |  |  |  |
| ^†^ Group 1: Young children with 0-1 target joint (cases no. 3, 7, 12)  Group 2: Adolescents with 2-3 target joints (cases no. 1, 2, 4, 5, 13) Group 3: Young adults with >3 target joints (cases no. 6, 8, 9, 10, 11) | | | | | | |
